# Pilot Clinical Trial of Time-Restricted Eating in Patients with Metabolic Syndrome

**DOI:** 10.3390/nu13020346

**Published:** 2021-01-24

**Authors:** Iwona Świątkiewicz, Celestyna Mila-Kierzenkowska, Alina Woźniak, Karolina Szewczyk-Golec, Jarosław Nuszkiewicz, Joanna Wróblewska, Paweł Rajewski, Simone J. P. M. Eussen, Kristine Færch, Emily N. C. Manoogian, Satchidananda Panda, Pam R. Taub

**Affiliations:** 1 Department of Cardiology and Internal Medicine, Collegium Medicum, Nicolaus Copernicus University, 85-094 Bydgoszcz, Poland; 2 Division of Cardiovascular Medicine, University of California San Diego, La Jolla, CA 92037, USA; ptaub@health.ucsd.edu; 3 Department of Medical Biology and Biochemistry, Collegium Medicum, Nicolaus Copernicus University, 85-092 Bydgoszcz, Poland; celestyna@o2.pl (C.M.-K.); alina-wozniak@wp.pl (A.W.); karosz@cm.umk.pl (K.S.-G.); jnuszkiewicz@cm.umk.pl (J.N.); jwroblewskapraca@gmail.com (J.W.); 4 Center for Obesity and Metabolic Disorders Treatment, 85-676 Bydgoszcz, Poland; rajson@wp.pl; 5 Department of Epidemiology and School for Cardiovascular Diseases (CARIM), Maastricht University, 6200 MD Maastricht, The Netherlands; simone.eussen@maastrichtuniversity.nl; 6 Steno Diabetes Center Copenhagen, 2820 Gentofte, Denmark; kristine.faerch@regionh.dk; 7 Department of Biomedical Sciences, University of Copenhagen, 2200 Copenhagen, Denmark; 8 Salk Institute for Biological Studies, La Jolla, CA 92037, USA; emily.manoogian@gmail.com (E.N.C.M.); panda@salk.edu (S.P.)

**Keywords:** time-restricted eating, metabolic syndrome, eating pattern, clinical trial, m-health applications, cardiometabolic risks, health outcomes, body weight, circadian rhythm, dietary assessment methodologies

## Abstract

Metabolic syndrome (MetS) and erratic eating patterns are associated with circadian rhythm disruption which contributes to an increased cardiometabolic risks. Restricting eating period (time-restricted eating, TRE) can restore robust circadian rhythms and improve cardiometabolic health. We describe a protocol of the Time-Restricted Eating on Metabolic and Neuroendocrine homeostasis, Inflammation, and Oxidative Stress (TREMNIOS) pilot clinical trial in Polish adult patients with MetS and eating period of ≥14 h/day. The study aims to test the feasibility of TRE intervention and methodology for evaluating its efficacy for improving metabolic, neuroendocrine, inflammatory, oxidative stress and cardiac biomarkers, and daily rhythms of behavior for such population. Participants will apply 10-h TRE over a 12-week monitored intervention followed by a 12-week self-directed intervention. Changes in eating window, body weight and composition, biomarkers, and rhythms of behavior will be evaluated. Dietary intake, sleep, activity and wellbeing will be monitored with the myCircadianClock application and questionnaires. Adherence to TRE defined as the proportion of days recorded with app during the monitored intervention in which participants satisfied 10-h TRE is the primary outcome. TREMNIOS will also provide an exploratory framework to depict post-TRE changes in cardiometabolic outcomes and behavior rhythms. This protocol extends previous TRE-related protocols by targeting European population with diagnosed MetS and including long-term intervention, validated tools for monitoring dietary intake and adherence, and comprehensive range of biomarkers. TREMNIOS trial will lay the groundwork for a large-scale randomized controlled trial to determine TRE efficacy for improving cardiometabolic health in MetS population.

## 1. Introduction

Metabolic syndrome (MetS) occurs in ~30% of adults and is associated with increased cardiometabolic risks [1,2,3,4,5]. The MetS is characterized by multiple risk factors for diabetes mellitus (T2DM) and cardiovascular disease (CVD) such as central obesity, elevated fasting plasma glucose (FPG), dyslipidemia, and elevated blood pressure (BP) [2,3,4,6]. Patient adherence and effectiveness of traditional structured lifestyle interventions that address disrupted metabolic homeostasis through low-calorie diets, increase in physical activity, and promotion of weight loss are low and not sustainable long term [7,8,9].

A daily rhythm in the eating–fasting cycle supports a robust circadian rhythm in metabolic processes involved in glucose homeostasis, protein synthesis, lipid synthesis and oxidation, and mitochondria function [10,11,12,13]. Misalignment between daily rhythms of food intake and the sleep–wake cycle and circadian timing system can contribute to circadian rhythm disruption, which results in a disruption of metabolic homeostasis, increased oxidative stress, activation of inflammation, and dysregulation of circadian-related hormones [13,14,15,16,17]. Circadian disruption is associated with an increased risk of obesity, MetS, T2DM, and CVD [14,18,19,20]. Erratic eating patterns such as eating over a prolonged period per day and irregular meal timing, which are often found in patients with MetS, are associated with circadian rhythm disruption and increased cardiometabolic risks [20,21,22,23,24,25,26,27].

Novel approaches which aim at restoring robust circadian rhythms through maintaining an appropriate daily rhythm of eating–fasting cycle represent a promising avenue for improving cardiometabolic risks [11,12,13,26,28]. Time-restricted eating (TRE) is a lifestyle intervention in which eating is restricted to a reduced, fixed number of hours (h) per day, which in various pathways promotes improved cellular metabolism [11,12,13,25,26,28]. In animal-based studies, TRE restores normal levels and/or normal daily rhythms in several mRNAs, proteins, and metabolites that are implicated in metabolic homeostasis of glucose, lipids, redox, and mitochondria function, and regulates circulating circadian-related hormones [11,12,13,26,29,30,31,32]. An implementation of 8–12-h TRE in animal models improves metabolic function by preventing fatty liver, dyslipidemia, and glucose intolerance, as well as results in improvements in sleep, body weight, motor coordination, cardiac health, and endurance level. TRE can impart benefits irrespective of nutrition quantity and quality and seems to be both preventive and therapeutic for cardiometabolic diseases [13].

TRE offers a beneficial strategy for patients with cardiometabolic disease [33]. One pilot TRE study (19 patients) provides results that are specific to American population with diagnosed MetS [34]. Several small-scale human studies of individuals with various metabolic disorders but without overt metabolic disease, as well as a feasibility study of obese patients with T2D, suggest usefulness of TRE for improving cardiometabolic health [25,35,36,37,38,39,40,41,42,43,44,45,46,47]. It has been demonstrated that TRE can be feasible and effective for weight loss and a decrease in body fat and energy intake, as well as improvements in glucose tolerance, insulin resistance, glycemic control, lipid levels, sleep, quality of life, and BP. However, previous TRE human studies were mostly focused on evaluating the feasibility and/or effects of TRE in healthy individuals with normal weight [48,49,50,51,52,53,54,55] and overweight or obese adults without overt metabolic disease [25,34,35,36,37,38,39,40,41,42,43,44,45,46]. The interpretation and generalization of the effectiveness of TRE in patients with MetS can be affected by a limited number of human studies, various populations, a lack of the diagnosed MetS as an inclusion criterion, small sample size, short-term duration of TRE intervention and follow-up, various TRE eating windows, limited spectrum of assessed biomarkers, and non-optimal tools for recording dietary intake and monitoring an adherence to TRE. There is only scant data on long-term adherence and sustainability of TRE intervention in subjects with metabolic disorders [25,34,35,43].

In addition, the effects of TRE on glycemic measures and lipids can be beneficial but data are limited and not uniformly consistent among different studies. For example, in some studies of overweight and obese subjects, a decrease in FPG as well as fasting, mean and nocturnal glucose levels obtained from continuous glucose monitor (CGM) was observed post-TRE [35,36,39,41,45]. In contrast, no improvement was also reported [34,37,38,40,46]. In the pilot study of patients with MetS and the study of obese subjects with prediabetes and elevated BP, TRE led to significant reduction of atherogenic lipids [34,39]; however, no significant benefits or even worsening of atherogenic lipids were also reported in subjects with MetS components [35,37,38,41]. It is also important to note that different studies used different protocols and methodologies, which may contribute to some differences in the reported results. In addition, the effects of TRE on other metabolic, neuroendocrine, inflammatory and oxidative stress biomarkers, and BP have been poorly investigated [34,37,38,41,44].

Data of the feasibility and effectiveness of TRE in European populations with metabolic disorders including patients with MetS are limited. In this article, we describe a protocol of the pilot clinical trial in Poland to investigate the impact of Time-Restricted Eating on Metabolic and Neuroendocrine homeostasis, Inflammation, and Oxidative Stress (TREMNIOS) in adult population of patients with MetS and prolonged eating period during a day. The TREMNIOS trial addresses the feasibility of TRE intervention which includes the assessment of adherence to TRE and myCircadianClock application (mCC app), recruitment procedures, and the suitability of methods for data collection. In addition, the TREMNIOS trial is expected to provide exploratory data depicting changes in cardiometabolic outcomes and daily rhythms of behavior related to TRE, and preliminary evaluation whether TRE is associated with improvements in cardiometabolic health and personal sense of wellness in Polish patients with MetS. The underlying hypothesis is that imposing eating–fasting cycle through TRE without changing nutrition quality and quantity or the intensity of physical activity will restore robust rhythmic daily behavior and improve cardiometabolic outcomes and overall health of patients with MetS.

## 2. Materials and Methods

### 2.1. Study Design

**Figure 1:**
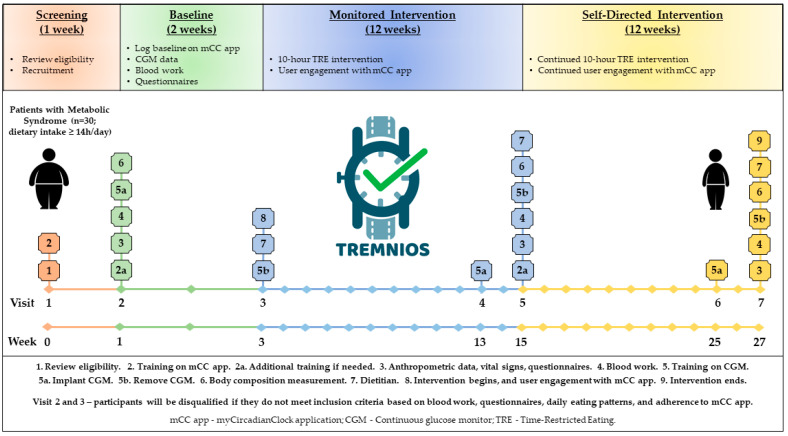
Study design.

TREMNIOS is a multicenter single-arm pilot clinical trial which will be performed in the Polish population of adult patients with MetS and eating period of ≥14 h per day. The essential components of the research plan and its timeline are depicted in Figure 1.

The study will consist of 1 week of screening, 2 weeks of baseline assessment, 12 weeks of monitored intervention, and 12 weeks of self-directed intervention. The participants will be asked to restrict their food intake to 10 h a day and fast for the remaining 14 h over 12 weeks of TRE monitored intervention. Eating (dietary timing and intake, and compliance with TRE), sleep, and physical activity patterns will be assessed throughout the study with a custom-made smartphone application (mCC app) [25]. During the TRE monitored intervention, participants will receive education and support, including daily prompts from the mCC app. Then, the participants will enter the 12-week self-directed TRE intervention during which they will continue the TRE intervention and use the mCC app to record all dietary intake, exercise, and sleep. During the self-directed intervention, participants will not receive feedback from study team members or the mCC app.

The primary study outcome that characterizes the feasibility of TRE intervention in population of patients with MetS is an adherence to TRE intervention that is defined as the proportion of total number of days recorded with mCC app during the TRE monitored intervention period in which participants satisfied a requirement of a 10-h TRE eating window. In addition to the adherence, other characteristics of the feasibility of the TRE program will be evaluated. These characteristics include screening failure rate (i.e., a number of persons screened compared to final enrollment), retention rate (i.e., a number of participants enrolled compared to the number of participants who finish the final study measures), reasons for screening failures and dropout from the study, as well as an adherence to mCC app (i.e., completion of mCCapp logs). The evaluation of this information will support the assessment of effectiveness of recruitment strategies and methods of monitoring adherence to TRE.

The secondary outcome measures include post-TRE changes in cardiometabolic outcomes, daily rhythms of behavior, and self-reported health. Glucose homeostasis will be evaluated with CGM for 2 weeks at the start of the study (baseline period), at the end of the monitored intervention, and the end of the self-directed intervention. The routine laboratory tests and various biomarkers from blood analysis, body weight and composition, as well as BP and heart rate (HR) will be assessed at baseline, at the end of the monitored intervention, and the end of the self-directed intervention. In parallel to these parameters, health questionnaires and chrono-nutrition questionnaire will be administered for self-reported health, wellness, as well as dietary and sleep timing assessment.

The study will be conducted in accordance with the Declaration of Helsinki. Approval from the Bioethics Committee of the Nicolaus Copernicus University in Toruń, Collegium Medicum in Bydgoszcz, Poland was obtained (KB 107/2019). All participants will provide informed consent. Approval from the Bioethics Committee of the Salk Institute was obtained for the use of the mCC app data (IRB 15-0003/2019). All participants will receive information about the terms and conditions of the use of the mCC app before providing informed consent. The study has been registered at www.clinicaltrials.gov (ClinicalTrials.gov, ID: NCT04328233, registered on 30 March 2020).

### 2.2. Subjects and Eligibility

A total of 30 participants with MetS and prolonged daily eating duration will be enrolled in the study. The inclusion and exclusion criteria are described in Table 1.

Participants must have FPG of ≥100 mg/dL and two additional indicators of MetS, self-reported dietary intake of ≥14 h per day, regular daytime schedule of activity, self-reported habitual sleep duration of >6.5 h, and a smartphone with Apple operating system (OS) or Android OS [4]. We chose to use FPG as one of the necessary inclusion criteria of MetS diagnosis for several reasons. It is known that high FPG and T2D are common and associated with an increased risk of CVD and mortality [4,56]. High FPG is a prevalent component of MetS-component clusters which are frequent and have an increased cardiovascular risk [1,57,58,59]. Circadian rhythm disruption is associated with abnormal glucose metabolism and it was shown that TRE can improve glucose regulation and maintain blood glucose level within homeostatic range [10,11,12,13,30].

The exclusion criteria include diagnosis of diabetes mellitus, shift work, planned travel over time zones, recent history of major adverse cardiac events, other active or uncontrolled medical conditions, history of eating disorder or bariatric surgery, participation in the weight-management program, special or prescribed diet for other reasons, substance abuse, depression, sleep apnea, and treatment with antidepressants, medication affecting glucose metabolism or appetite, or immunosuppression.

### 2.3. Screening and Recruitment

Participants will be recruited from the clinics at the Collegium Medicum, Nicolaus Copernicus University, Bydgoszcz, Poland (CM), and at the Center for Obesity and Metabolic Disorders Treatment, Bydgoszcz, Poland (COMDT). Participants must satisfy eligibility criteria (Table 1). An initial screening to determine the eligibility of potential participants will be performed by reviewing patient medical records and checking interest in the study by phone, or by interviewing potential participants during office visits at the clinics. Following an initial screening, potential subjects will be invited to visit the Cardiology Clinic at the CM or the COMDT to provide detailed information about the study, verify the eligibility, obtain informed consent, and complete baseline measurements.

At Visit 1 (Week 0—screening), a detailed interview will be conducted to further determine if the subject meets the inclusion and exclusion criteria (Figure 1). Participants will undergo a standard physical examination to verify the eligibility for the study. A physician will review the informed consent form with each participant, including clarification of possible questions; individuals still interested in participating in the study will sign the consent form in two copies, one to be given to the participant, and one to be stored with the study documents in a locked cabinet in a secure room at the Department of Medical Biology and Biochemistry at the CM. General health and chrono-nutrition questionnaires will be administered to participants. The questionnaires should be completed within one week after Visit 1. The Beck Depression Inventory-II (BDI-II) will be used to screen for depression, the Epworth Sleepiness Scale (ESS) will be used to exclude for sleep apnea. Visit 1 will also entail an introduction to the mCC app.

### 2.4. Baseline Assessment

At Visit 2 (Week 1—baseline), which will be held in the Department of Medical Biology and Biochemistry at the CM, participants who are eligible based on the health questionnaires will be subject to further steps of the trial. The anthropometric data and vital signs including HR and BP will be collected. Visit 2 will also entail a fasting blood work. All participants will have their blood drawn by a certified laboratory diagnostician. The baseline laboratory tests will include the following: comprehensive metabolic panel (CMP), complete blood count (CBC), and various biomarkers (metabolic, neuroendocrine, inflammatory, and oxidative stress) (see Section 2.6.3. for further details). Additional training on the mCC app will be performed if needed. Participants will be asked to log their habitual dietary intake and timing of physical activity and sleep on the mCC app for 2 weeks. They will also have CGM (Abbott Freestyle Libre Pro) to continuously record blood glucose for 2 weeks. The participants will also undergo a body composition measurement (with a body composition analyzer using bioelectric impendence technology).

### 2.5. TRE Intervention

At Visit 3 (Week 3—intervention begins), participants will return to remove the CGM and review their mCC app data. Participants who are eligible based on blood work results and logging mCC app data will be subject to further steps of the trial. The food intake data from the baseline period will be downloaded from the server-side of the mCC app and the review of the data will be performed. Participants who satisfy logging criteria (recording ≥3 meals or beverages daily for at least 5 days a week) and have an eating interval of ≥14 h a day will undergo further approach. Participants who record <2 meals/day for >3 days or those with <6.5 h self-reported sleep for >3 days based on their data from the mCC app will be excluded from the study. The participants who meet the criteria for MetS (based on blood work results, BP, and/or waist circumference measurements), the mCC app logging criteria, and the BDI-II and ESS questionnaire criteria will be enrolled in the study (Table 1).

At Visit 3, participants will enter a 12-week monitored intervention between Weeks 3 and 15. Participants will meet with a dietitian in-person for behavioral nutritional counseling. At this visit, participants will be asked to restrict their food intake daily to 10 h a day and fast for 14 h. They will also be instructed to start the TRE intervention by selecting (together with study investigator) a 10-h eating window that best suits their lifestyle based on his/her baseline eating pattern from the first 2 weeks. This 10-h window must be between 7 a.m. and 9 p.m., with the last meal (including non-water beverage) consumed at least 2 h prior to the typical bedtime. The 10-h interval will be entered in the app, so participants can visualize their chosen daily eating window and consume all meals within this interval. Beverages that include caffeine or artificial sweeteners are not allowed outside of the 10-h feeding period, as some artificial sweeteners may affect glucose tolerance and caffeine can alter metabolism. Participants will also be able to modify the 10-h window based on their schedule; however, eating outside of the eating window by more than one hour more than twice per week is not allowed. TRE will be the only intervention, and participants will not be instructed to change their habits regarding physical activity or the quality, quantity, or caloric content of their diet.

During the 12-week monitored intervention, participants will continue to use the mCC app to document all dietary intake, exercise, and sleep quantity/quality. They will record their dietary intake by taking a picture of a food meal and/or beverage and/or annotating the food (with the food name, portion size, etc.). The mCC app will be programmed so that participants can visualize their caloric intake spread over the wakeful hours. Participants will receive push notifications (2–3 times a week) to provide information about TRE and metabolic health. The mCC app can be set by participants to alert them 30 min prior to the end of the eating interval to finish their last meal of the day. At Visit 4 (Week 13), which is 10 weeks into the intervention period, the participants will return to the clinic to have the CGM device placed for 2 weeks. At Visit 5 (Week 15), participants will visit the clinic to remove the CGM. At this visit, they will also have a fasting blood draw, vital signs assessment, body composition measurement, and be asked to complete the same general health and chrono-nutrition questionnaires as at Visit 1.

After 12 weeks of monitored intervention, participants will enter a 12-week self-directed intervention for up to a 6-month point. Beyond Week 15, participants will continue to use the mCC app to document all dietary intake, exercise, and sleep quantity/quality. During the 12-week self-directed intervention, no feedback from study investigators or push notifications from the mCC app will be provided to participants. At Visit 6 (Week 25), participants will return to the clinic for the application of CGM for 2 weeks. At Visit 7 (Week 27—intervention ends), participants will visit the clinic to remove the CGM. At this visit, they will also have a fasting blood draw, vital signs assessment, body composition measurement, and be asked to complete the same general health and chrono-nutrition questionnaires as at Visit 1.

### 2.6. Measurements

#### 2.6.1. Anthropometry

The anthropometric measurements will be performed at Visits 2, 5, and 7. Participants will have their weight and height measured in the fasted state on a digital scale with a height rod. The accuracy of the measurements is 0.1 kg and 0.5 cm, respectively. Body mass index (BMI) will be calculated using the formula [weight(kg)/height^2^(m^2^)] and the cut-off points of the World Health Organization will be used. Waist circumference will be measured in a fasted state immediately above the iliac crest using an anthropometric tape accurate to 0.5 cm. Changes in body weight, BMI, and waist circumference between Visits 2, 5, and 7 will be considered as the secondary outcome measures. Change in body weight between Visits 2 and 5 will be considered as the main secondary outcome measure.

#### 2.6.2. Continuous Glucose Monitor

All participants will be fitted with a CGM, and be instructed on its use. The Abbott Freestyle Libre Pro CGM will be used which has been validated for 14 days of continuous use with factory calibration. CGMs estimate blood glucose levels with high accuracy that correlates with those obtained from either venous or capillary blood. CGM will measure interstitial fluid glucose every 15 min for 14 full days, using a subcutaneous sensor placed in the upper arm area. The participant will wear the CGM for 2 weeks during the baseline period, at the last 2 weeks of the 12-week monitored intervention, and the 12-week self-directed intervention. Participants will be blinded to their CGM data during the entire study. Changes in the mean daily glucose level, post-prandial glucose response, mean amplitude of glycemic excursion, continuous overall net glycemic action, and the glycemic variability index will be calculated.

Changes in mean daily glucose levels obtained by CGM between the baseline period, the last 2 weeks of the 12-week monitored intervention, and the last 2 weeks of the 12-week self-directed intervention will be considered as the secondary outcome measures. Changes in mean fasting glucose levels obtained by CGM between Visits 2, 5, and 7 will be considered as the secondary outcome measures.

#### 2.6.3. Blood Samples

All participants will have their blood drawn at Visits 2, 5, and 7 in the morning after overnight fasting. Measurements of routine laboratory tests such as CMP including FPG, calcium, urea nitrogen, creatinine, sodium, potassium, chloride, total protein, serum albumin, bilirubin, alkaline phosphatase, aspartate aminotransferase, and alanine aminotransferase, as well as thyroid-stimulating hormone, glycated hemoglobin (HbA1c), and lipid profile including total cholesterol (TC), low-density lipoprotein (LDL)-cholesterol, high-density lipoprotein (HDL)-cholesterol, and triglycerides (TG), will be done in serum samples in a certified analytical laboratory.

Measurements of metabolic, neuroendocrine, inflammatory, and oxidative stress/antioxidant defense biomarkers other than listed above will be performed in the Department of Medical Biology and Biochemistry at the CM. Blood samples will be collected into polypropylene tubes (6 mL) without anticoagulant to obtain serum or in ethylenediamine tetraacetic acid (EDTA)-containing tubes (9 mL) to obtain plasma and erythrocytes. All samples will be centrifuged (6000× *g* for 10 min at 4 °C). The serum and plasma will be separated and stored at −80 °C for further analysis. Subsequently, the erythrocytes will be washed three times with phosphate-buffered saline (PBS) solution at a ratio of 1:3 with simultaneous centrifugation of the sample after each wash (6000× *g* for 10 min at 4 °C). The washed red blood cells will be mixed with a PBS solution to obtain erythrocytic suspension with a 50% hematocrit index. The suspension will be used to determine the parameters of oxidative stress.

Metabolic and neuroendocrine markers will include but will not be limited to free fatty acids (FFA), insulin, insulin-like growth factor-1 (IGF-1), resistin, irisin, ghrelin, visfatin, omentin-1, adiponectin, leptin, cortisol, and melatonin. The degree of insulin resistance will be estimated by homeostasis model assessment (HOMA). In particular, an insulin resistance score (HOMA-IR) will be computed with the formula: FPG (mmol/l) times fasting insulin (mU/L) divided by 22.5. High HOMA-IR values indicate low insulin sensitivity referred to as insulin resistance. Inflammatory biomarkers will include but will not be limited to high sensitivity (hs) C-reactive protein (CRP), hs interleukin (IL)-6, IL-8, IL-10, tumor necrosis factor-α (TNF-α), tumor growth factor-β1 (TGF- β1), growth/differentiation factor 15 (GDF 15), and lectin-type oxidized LDL receptor 1 (LOX-1). At present, CRP is the preferred biomarker of inflammation for cardiovascular risk stratification [60]. Oxidative stress/antioxidant defense markers will include but will not be limited to superoxide dismutase-1 (SOD-1), catalase (CAT), glutathione peroxidase (GPx), oxidized LDL (oxLDL), thiobarbituric acid reactive substances (TBARS), conjugated dienes (CD), malondialdehyde (MDA), 4-hydroxynonenal (4-HNE), vitamin A, and vitamin E.

The commercially available multiplexed enzyme-linked immunosorbent assay (ELISA) panels will be used for most metabolic, inflammatory and neuroendocrine markers, as well as oxLDL and 4-HNE measurements. ELISA tests will be run in triplicate along with standard dilution series for quantification following manufacturer’s instruction. The oxidative stress/antioxidant defense biomarkers will be assayed by specific methods described in detail elsewhere [15]. In brief, the levels of TBARS will be assayed according to the methods of Buege and Aust with the modification of Esterbauer and Cheeseman. The level of CD and the activities of SOD-1, CAT, and GPx will be determined according to Sergent et al., Misra and Fridovich, Beers and Sizer, and Paglia and Valentine, respectively. High-performance liquid chromatography (HPLC) system will be used to determine the levels of vitamins A and E and the MDA concentrations according to the methods of Wielinski and Mateos et al., respectively.

Changes in metabolic, neuroendocrine, inflammatory, and oxidative stress biomarkers measurements between Visits 2, 5, and 7 will be considered as the secondary outcome measures. Change in FPG measurements between Visits 2 and 5 will be considered as the main secondary outcome measure.

#### 2.6.4. myCircadianClock Application

The mCC app is a free smartphone application that was developed at the Salk Institute for Biological Studies, La Jolla, CA 92037, USA [25]. S.P. and E.N.C.M. from the Salk Institute, who are also members of the research team of this study, are the administrators of data collected on the server using this application.

The mCC app will serve as an electronic food, activity, and sleep diary. During the TRE intervention, data from the mCC app will be used to assess participant adherence, eating window, physical activity, and sleep. The mCC app is designed to run on Android and iOS devices and uses HIPAA compliant Amazon Web Server for server-side operations. A dedicated team of developers makes periodic updates to the app and to the backend server to comply with updates released by Apple, Google, and Amazon. The server side is designed to run multiple independent studies and the app is designed for individual customization. This allows study-specific customization by the investigator and user-specific customizations by participants.

For the TREMNIOS clinical trial, the mCC app was customized to be used in a single group assignment (TRE intervention group) study. After the enrollment of participants at Visit 2, the study investigator will generate a unique activation code. During the 2-week baseline period, participants will be asked to log their habitual food intake, physical activity, and sleep. Participants will not receive unsolicited feedback from the study investigators or the mCC app during baseline. At Visit 3, subjects will self-select a 10-h interval for consuming all of their food and beverages during the TRE intervention. Their chosen eating interval is highlighted in the app so that they can easily track the progress of their daily eating within their set interval. Participants will be asked to log all food intake, physical activity, and sleep quantity/quality every day for the baseline period and for the duration of the TRE intervention between Visits 3 and 7 (Figure 1). Other details regarding the use of the mCC app during the TRE intervention are included in Section 2.5.

On the server-side, a sub-study dashboard was created for this clinical trial. In the study summary dashboard, the participant’s study code, and the date of activation of the app will be shown along with their daily logs in real-time. The designated members of the research team will have password-protected access to the real-time data, will log in to the dashboard at least twice weekly to monitor food intake data, and perform real-time tracking of TRE adherence, and follow up participants with inconsistent logging as necessary. If participants do not log or show inconsistent logging for two or more days, they will be contacted by telephone, and app users will be reinforced. Logging adherence will be determined by a minimum of two caloric entries >5 h apart for a given day. Adherence to the designated eating window implies that all caloric items were contained within a 15-min buffer on each side of the self-selected 10-h eating window. The time-stamped food data is also shown as a “feedogram” raster plot along with the eating duration for each day. This will offer a visual summary of the participant’s eating pattern. The protocol of this study has an app-based component allowing for real time tracking of adherence and correlating glucose levels with food intake.

The results from previous TRE studies which used mCC app [25,34,35] have demonstrated that adherence to mCC app use is high, mainly due to the simplicity of logging food metadata. The mCC app takes ~10 s for logging one meal by using the picture feature. If, for any reason, the participant does not wish to record a picture of food item(s), there will be also an option to enter the name of food in Polish or English (by choosing the name of food from the list) and the approximate time that food was consumed. Because a Polish version of mCC app is not available, we will provide participants with the list of common food with the names both in Polish and English. We will also provide the participants with required support regarding the use of mCC app, including special app training at Visits 1, 2, and 5. If a participant faces difficulty logging data or has questions about the app, he/she will be able to contact the study investigator through the feedback feature of the app. Although self-reporting of adherence by patients is generally not accurate, it was shown that the false-negative rate (i.e., food consumed but not logged) using the mCC app is ~10% [25,61].

The proportion of the total number of days recorded with the mCC app during the TRE monitored intervention period in which participants satisfied a requirement of a 10-h eating window will be used to quantify the adherence to TRE intervention. This proportion is considered as the primary outcome measure. Changes in eating window duration, defined as the duration from the first to last caloric intake over 24-h cycle collected via the mCC app, assessed at Visits 3, 5, and 7 will be considered as the secondary outcome measures. Changes in physical activity and sleep duration collected via the mCC app, assessed at Visits 3, 5, and 7 will be considered as the secondary outcome measures.

The food intake data (photo and/or annotation entries) will be downloaded from the server-side of the app and dietary analyses will be performed by a registered dietitian to calculate the overall calorie intake and estimated calories from macronutrient component [34]. Three-day food records from each study period (from baseline and the last 12 days of each phase of TRE intervention) will be randomly chosen and analyzed to characterize the macronutrient composition of the diet. Caloric estimates will be made using the Caloric King database. A dietitian will review the record with the participant to clarify potential omissions and ambiguities and to assure that additional information is provided to improve the accuracy of the macronutrient composition of the diet. Changes in calorie intake assessed at Visits 3, 5, and 7 will be considered as the secondary outcome measures. We note that a 3-day dietary intake protocol with no regard to whether the food intake occurs on weekdays or weekend may involve a limitation but is consistent with a protocol used by Wilkinson et al. [34] in the study of the US patients with MetS. Wilkinson et al. [34] indicated that their findings associated with an assessment of post-TRE calorie intake were consistent with other studies [25].

#### 2.6.5. Body Composition Analysis

The body composition measurements will be performed at Visits 2, 5, and 7 using bioelectrical impedance technology with the Tanita Scale DC 430U (Tokyo, Japan). Fat mass percentage, visceral fat rating, and greater muscle mass percentage will be evaluated. Changes in fat mass percentage between Visits 2, 5, and 7 will be considered as the secondary outcome measures. It is expected that in the follow-up larger randomized controlled trial, a more accurate dual-energy X-ray absorptiometry (DXA) method will be used, which was not possible in this pilot trial.

#### 2.6.6. Cardiac Parameters

Systolic and diastolic BP and HR measurements will be done at Visits 2, 5, and 7 under resting (after a 5-min rest) and fasting conditions and will result in an output that was an average of three readings 1–2 min apart. Changes in systolic BP, diastolic BP, and HR between Visits 2, 5, and 7 will be considered as the secondary outcome measures.

#### 2.6.7. Questionnaires

General health questionnaires and chrono-nutrition questionnaires will be collected at Visits 2, 5, and 7. The required permissions from the developers of the questionnaires for the use of non-commercial research purposes were obtained.

The ESS will be used to assess self-reported sleepiness. The BDI-II will be used for measuring depression severity. Sleep quality will be analyzed through the Pittsburgh Sleep Quality Index (PSQI). The Munich Chronotype Questionnaire (MCTQ) will be used to determine the self-reported chronotype. The self-reported health survey (SF-36 health survey) will be used to evaluate self-reported overall health and wellbeing. Regarding the eating disorder examination, the physician at Visit 1 and study investigator at Visits 2 and 3 will examine and interview the patients to determine whether any symptoms of an eating disorder occur. If the symptoms are detected the patient will not participate.

The chrono-nutrition questionnaire will be used to evaluate the habitual timing of dietary intake (frequency, (ir)regularity, and time window) during weekdays and weekends. Participants will be asked to fill in specific clock time points (30-min range) of getting up, eating and drinking (breakfast, lunch, dinner and in-between-snacks). Frequency of caloric intake will be calculated by summing up the number of eating and drinking clock time points. The time window of food and drink consumption will be determined by calculating the difference between the first and last episode when participants ate or drank something. Face validity was assessed and showed that completing the questionnaire takes approximately 10 min, and participants perceived the questionnaire easy to complete.

### 2.7. Primary and Secondary Outcome Measures

The primary and secondary outcome measures are depicted in Table 2. The adherence to TRE intervention is the primary outcome defined as the proportion of the total number of days recorded with mCC app during the TRE monitored intervention period (i.e., between Visit 3 and Visit 5) in which participants satisfied a requirement of a 10-h TRE eating window. The secondary outcome measures include changes in body weight, BMI, waist circumference, fat mass percentage, mean daily glucose levels obtained by CGM, mean fasting glucose levels obtained by CGM, FPG, lipids and HbA1c levels, HOMA-IR, as well as metabolic, neuroendocrine, inflammatory and oxidative stress biomarkers levels, eating window duration and timing of eating collected via mCC app and chrono-nutrition questionnaire, calorie intake collected via mCC app, physical activity and sleep duration collected via mCC app, systolic and diastolic BP, HR, and the health questionnaires scores. The main secondary outcome measures include changes in body weight and FPG between Visit 2 and Visit 5. The time frames for changes in all outcomes are indicated below in Table 2.

### 2.8. Data Collection and Management

Data will be collected by TREMNIOS trial team members with training and experience in clinical assessment. All data related to this study will be stored with the highest possible level of security. Completed data forms or other hard-copy documents containing protected health information will be kept in a locked file in the coordinator’s office at the Department of Medical Biology and Biochemistry, CM. Data will be entered into an electronic deidentified database (for subsequent transfer into SPSS for reporting and statistical analysis) by authorized team members trained in data management; participants will be identified only using a unique number. Electronic data will be stored on a secure server which is maintained, with regular back-ups, by CM system administrators and accessible from password-protected computers in the Department of Medical Biology and Biochemistry, CM. The principal investigator (I.S.) and designated team members will review all data collection forms on an ongoing basis for data completeness and accuracy as well as protocol compliance. Access to data with identifiers will be restricted to authorized team members and regulatory authorities. Any data, forms, and other records with identifiers that leave the site will be transported in a locked file box to maintain confidentiality. Identifiable data will be destroyed 5 years after study completion or 3 years after the last publication based on when the data is published, whichever comes last (unless future regulations dictate that the data be kept longer).

### 2.9. Statistical Analysis

Data will be analyzed using SPSS Statistics software (IBM Inc., Armonk, NY, USA). The normality of distribution will be examined for all variables and those found to have a non-normal distribution will be properly transformed before further analysis. Paired samples t-tests will be used to compare baseline to post-intervention value. The adherence to TRE intervention expressed as the percentage of days with achievement of required 10-h eating window during the TRE monitored intervention will be calculated per participant, and then reported as a group mean with standard deviation. A two-tailed *p* < 0.05 will be considered statistically significant.

A sample size calculation was performed which indicated that the TREMNIOS trial has a sufficient statistical power to detect clinically relevant changes in the two main exploratory outcomes, i.e., body weight and FPG. Specifically, using the GPower software (v.3.1.9.7) [62], we calculated that a sample size of 26 (which is consistent with ~10% dropout rate) provides 80% power at the 0.05 significance level to detect an effect size of 0.8 for the main exploratory outcomes of TREMNIOS trial. We note that this effect size is smaller than the previously observed TRE-induced body weight change [34,38,44] and fasting plasma glucose change [35,39,41,50].

## 3. Results

The TREMNIOS trial is currently recruiting participants. The estimated primary completion date of the study is July 2022. The TRE intervention is expected to improve cardiometabolic markers and daily rhythms of behavior in patients with MetS and prolonged daily eating period.

## 4. Discussion

To our knowledge, the TREMNIOS pilot clinical trial in Poland is the first study targeted at collecting comprehensive data on the feasibility and exploratory data on the effectiveness of TRE for improving cardiometabolic health and daily rhythms of behavior in the European adult population of patients with MetS and prolonged daily eating period. The protocol of TREMNIOS trial extends the previous TRE-related protocols by targeting European population of patients with diagnosed MetS and including longer duration of TRE intervention, validated tools for recording food intake and monitoring an adherence to intervention, as well as more comprehensive range of metabolic, neuroendocrine, oxidative stress and inflammatory biomarkers. The study will lay the essential groundwork for a planned large-scale randomized controlled trial to determine the effectiveness and sustainability of TRE for reducing long-term cardiometabolic risks in patients with MetS.

The rationale for the TREMNIOS trial builds largely on extensive mechanistic molecular and physiological studies of circadian rhythms, eating patterns, and metabolic homeostasis, both in animal models and human studies, as well as the findings of several previous small-scale TRE human trials. Although TRE provides a promising strategy for the prevention and treatment of cardiometabolic diseases, evidence on the effectiveness of TRE in patients with MetS is limited, thus further focused studies are needed. Importantly, there is a paucity of information about the feasibility and long-term sustainability of an implementation of TRE as an alternative approach in the management of the European populations of patients with MetS, and there is limited data on effects of TRE on cardiometabolic outcomes in these populations.

Several small-scale TRE studies in healthy humans with normal weight demonstrated that TRE can be feasible, safe, and beneficial dietary strategy [48,49,50,51,52,53,54,55]. TRE resulted in a decrease in energy intake [48,51], body weight even without reducing calorie intake [48,49], body fat even without impacting lean mass [49,50,52,54], BP [49], blood glucose and TG [50], glucose intolerance [55], leptin and inflammatory markers levels [50], and feeling of hunger [55], as well as an increase in adiponectin and HDL-cholesterol levels [49,50], muscular strength, and endurance capacity [51,52,55]. Although no significant impact of TRE on body weight [50,51], body fat [51], glucose and insulin levels [49,52], some lipids [49,50,52], inflammatory markers [55], and cortisol pattern [49,52] or even worsening of glucose metabolism measures [53] were observed in several studies of healthy subjects, available data indicated that TRE can be effective for improving cardiometabolic outcomes.

To our knowledge, one pilot study of an American population of patients with diagnosed MetS [34] and several small-scale TRE trials in the populations (mainly from the US) with elevated BMI (overweight or obese subjects) [25,35,36,37,38,40,41,42,44,45,46], increased waist circumference (individuals with central obesity) [39,40,43], and disorders of glucose metabolism (subjects with prediabetes, high risk of T2DM, and T2D with controlled hyperglycemia (HbA1c < 9%) either by diet or taking ≤ two oral hypoglycemic agents) [37,39,47] were conducted. The trials of individuals with metabolic disorders demonstrated that TRE result in several cardiometabolic benefits such as weight loss [25,34,35,38,39,40,43,44] and a decrease in body fat [34,35,36,43,44] and energy intake [25,34,36,38,44,47], as well as improved glucose tolerance [39], insulin resistance [37,38,41,44], glycemic control [35,36,39,41,43,45], lipid levels [34,39], sleep [25], quality of life [25,40,45], and reduced BP [34,37,38]. In the pilot study of American patients with MetS, the improvements in body weight and fat, waist circumference, atherogenic lipids, and BP were observed despite no overt attempt to change diet quantity and quality or physical activity [34]. Moreover, previous TRE trials of subjects with metabolic disorders reported good adherence with TRE for a period up to 16 weeks [25,34,35,38,43,47].

Despite the encouraging results of previous TRE human studies, various aspects associated with an implementation of TRE in patients with diagnosed MetS require further clinical research which should be conducted exclusively in such populations of patients. Specifically, long-term feasibility of TRE intervention deserves further attention. In the pilot study of American population with MetS, the adherence to TRE and mCC app during a 12-week period was shown to be ~90 and ~85%, respectively [34]. In the study of diabetic patients, the rate of adherence varied greatly from 4% to 100% even over a relatively short period of 4-week TRE intervention [47]. This indicates that the adherence to TRE can be variable and is not always satisfactory. Wilkinson et al. [34] also observed that self-selecting of TRE eating window may be conducive to better adherence to TRE intervention. However, data on the extent to which MetS patients maintain a shortened daily eating window for an extended period of time during TRE intervention is lacking. The TREMNIOS trial is expected to provide insights into this question by evaluating the adherence of MetS patients to TRE over a relatively long period of 24 weeks. The TREMNIOS data characterizing the feasibility will aid in the design of a larger randomized controlled trial.

The impact of TRE on weight loss, decrease in body fat, energy restriction, decreasing appetite, and delimitation of eating window as well as the interactions between these TRE effects require elucidation. In addition, the effects of TRE on glycemic measures and lipid levels require further investigation as available data are limited and highly variable. Specifically, in several studies, no significant TRE-related improvements of some glucose or lipid measures were observed [34,35,36,37,38,39,40,43,45,46,47]. In addition, some findings suggest an increase in lipids post-early TRE or elevated FPG and morning glucose intolerance post-late TRE [37,41,53]. Moreover, TRE effects on metabolic, inflammatory, and oxidative stress biomarkers have been poorly investigated, so further evidence is needed regarding potential post-TRE improvements in oxidative stress, inflammation, and atherosclerosis [37,41,44,50,60]. In addition, more data on potential benefits of TRE on circadian-related metabolic and neuroendocrine biomarkers, BP, and daily rhythms of behavior such as eating window, meal timing, sleep, and physical activity would be desirable [63,64,65].

In addition to targeting an European population of patients with MetS, the protocol of TREMNIOS trial extends the protocols used in recent and ongoing TRE-related studies [25,34,35,38] by including exclusively patients with diagnosed MetS, longer duration of TRE intervention (of 24 weeks), more comprehensive range of various cardiometabolic biomarkers including circadian-related hormones, analyses of biomarkers at multiple time points over a period of 6 months, application of CGM for an evaluation of glucose control, use of mCC app as the validated method for recording eating pattern and nutrient intake, sleep, activity and monitoring of an adherence to TRE, as well as additional tools for chrono-nutrition assessment. These extensions are important to advance an understanding of the roles of metabolic regulatory mechanisms and circadian system in the pathophysiology of MetS and its complications. Available data seem to support a 10 h TRE eating window that has been chosen for this trial. This window still produces benefit and long-term adherence is expected to be better compared to the application of more restrictive eating period [25,34]. The multi-approach methodology will provide a means to comprehensively evaluate the feasibility of TRE and provide an exploratory data on the impact of TRE on eating pattern, nutrient intake, metabolic homeostasis, and overall health of the population of patients with MetS during the 6-month TRE intervention.

The TREMNIOS trial is expected to lay the essential groundwork for a planned large-scale randomized controlled trial to determine if adopting a simple TRE pattern intervention can lead to restoring rhythmic daily behavior resulting in a reduction of cardiometabolic risks and improvements of health status in Polish population of patients with MetS and prolonged daily eating period. The results of this trial will be useful for a design of future large-scale trial in terms of sample size, inclusion/exclusion criteria, duration and timing of TRE eating window, length of TRE intervention, primary and secondary outcomes, use of the mCC app, and spectrum of biomarkers for evaluating the efficacy of TRE intervention in patients with MetS. TRE studies on the American populations of patients with MetS have been recently undertaken in the US under the leadership of scientists who are the members of TREMNIOS research team (P.R.T., S.P., and E.N.C.M.). The initiation of acquisition of TRE-related data for the European population of patients through the TREMNIOS trial and the use of consistent protocols will facilitate creation of joint databases and comparative analysis of patient populations with MetS from different countries and continents.

The TREMNIOS trial will examine one group of 30 patients (pre-post TRE intervention) with no control group which is a potential limitation. This approach has been chosen given that this trial is a precursor for large-scale controlled trial in the future and available funding at this initial stage of study is limited which limits the overall number of patients. Following the results of power analysis, it is deemed advantageous to conduct a single arm trial with 30 participants rather than the controlled trial with a smaller sample size of each group. Whereas the evaluation of the adherence to the TRE intervention does not require a control group, the interpretation of exploratory data on cardiometabolic parameters will require caution. For example, because there is no control group, one cannot exclude the possibility that TRE-related changes in cardiometabolic parameters will be to some extent affected by the process of monitoring during the TRE intervention. The planned randomized controlled trial to be conducted after the TREMNIOS trial is expected to be free of this limitation. However, the lack of a control group in the TREMNIOS trial may limit the ability to predict the acceptability of participants to be randomized to intervention and control groups in a planned large-scale randomized-control trial.

## 5. Conclusions

The protocol of the TREMNIOS clinical trial addresses the application of TRE intervention in the Polish population of patients with MetS. The protocol has been designed to test the long-term feasibility of TRE intervention and use of mCC app, and provide exploratory data on the effects of TRE on cardiometabolic health by including a comprehensive suite of circadian rhythm-related measurements of dietary patterns, metabolic and neuroendocrine homeostasis, inflammation, oxidative stress and cardiac markers, sleep, activity, and overall sense of wellness. The findings of this pilot clinical trial will provide a basis for a planned large-scale randomized controlled trial to determine the efficacy and sustainability of TRE intervention for reducing long-term cardiometabolic risk, providing tools for sustained lifestyle changes and, ultimately, improving overall health-related markers in patients with MetS.

## Figures and Tables

**Table 1:** Eligibility criteria.

**Inclusion Criteria**
Age 18–75 years Body mass index ≥25 kg/m^2^ Metabolic syndrome defined as elevated fasting plasma glucose ≥100 mg/dL AND two or more of the following criteria increased waist circumference ≥102 cm in men, ≥88 cm in women elevated fasting plasma triglycerides ≥150 mg/dL (or on drug treatment for elevated triglycerides) reduced high-density lipoprotein (HDL)-cholesterol <40 mg/dL for men, <50 mg/dL for women (or drug treatment for reduced HDL-cholesterol) elevated blood pressure, systolic blood pressure ≥130 mm·Hg and/or diastolic blood pressure ≥85 mm·Hg (or drug treatment for hypertension) Own a Smartphone with Apple operating system (OS) or Android OS Average eating period ≥14 h/day Habitual sleep duration of >6.5 h If patients are on cardiovascular medications (such as lipid-modifying drugs including statins, over the counter drugs such as red yeast rice and fish oil, or anti-hypertensive drugs), no dose adjustments will be allowed during the study period
**Exclusion Criteria**
Diagnosis of diabetes Pregnant or lactating women Active smoking or illicit drug use or history of treatment for alcohol abuse Shift work Caregivers for dependent requiring nocturnal care Planned travel over one time zone during the study period History of a major adverse cardiovascular event within the past one year (acute coronary syndrome, percutaneous coronary intervention, coronary artery bypass graft surgery, hospitalization for congestive heart failure, stroke/transient ischemic attack) or current uncontrolled arrhythmia Uncontrolled medical conditions due to rheumatologic, hematologic, oncologic, infectious, gastrointestinal, psychiatric, nephrological, or endocrine diseases Known history of an eating disorder Currently enrolled in a weight-loss or weight-management program On a special or prescribed diet for other reasons (e.g., celiac disease) Current treatment with antidepressants, medication affecting glucose metabolism or appetite, or immunosuppression History of bariatric surgery A score of >16 on the Epworth Sleepiness Scale Depression determined by the Beck Depression Inventory Failure to use the smartphone app for documentation during a 2-week baseline period

**Table 2:** Outcome measures.

**Primary Outcome Measures**
Adherence to TRE intervention defined as the proportion of the total number of days recorded with the myCircadianClock app in which the participants satisfied a requirement of a 10-h eating window ^1^
**Secondary Outcome Measures**
Body weight ^2,3,4^ Fasting plasma glucose ^2,3,4^ Body mass index ^2,3,4^ Waist circumference ^2,3,4^ Fat mass ^2,3,4^ Mean glucose level as measured by continuous glucose monitor ^2,3,4^ Mean fasting glucose level as measured by continuous glucose monitor ^2,3,4^ Lipids ^2,3,4^ Glycated hemoglobin ^2,3,4^ Homeostasis model assessment-insulin resistance score (HOMA-IR)^2,3,4^ Metabolic and neuroendocrine biomarkers ^2,3,4^ Inflammatory biomarkers ^2,3,4^ Oxidative stress/antioxidant defense biomarkers ^2,3,4^ Systolic and diastolic blood pressure ^2,3,4^ Heart rate ^2,3,4^ Calorie intake collected via the myCircadianClock app ^1,3,5^ Timing of dietary intake collected via the myCircadianClock app and chrono-nutrition questionnaire ^1,2,3,4,5^ Eating window duration collected via the myCircadianClock app and chrono-nutrition questionnaire ^1,2,3,4,5^ Physical activity duration collected via the myCircadianClock app ^1,3,5^ Sleep duration collected via the myCircadianClock app ^1,3,5^ Self-reported sleepiness as assessed from the Epworth Sleepiness Scale ^2,3,4^ Self-reported depression determined by the Beck Depression Inventory-II ^2,3,4^ Self-reported sleep quality as assessed from the questionnaire Pittsburgh Sleep Quality Index ^2,3,4^ Self-reported chronotype as assessed from the Munich Chronotype Questionnaire ^2,3,4^ Self-reported overall health and wellbeing as assessed from the self-reported health questionnaire (SF-36 health survey) ^2,3,4^

Each listed secondary outcome measure should be understood as a change in the measure over time-restricted eating (TRE) intervention period. Time frames for changes: ^1^ between Visits 3 and 5; ^2^ between Visits 2 and 5; ^3^ between Visits 5 and 7; ^4^ between Visits 2 and 7; ^5^ between Visits 3 and 7 (see Figure 1 for details).

## Data Availability

Not applicable.
